# Finger Thickening during Extra-Heavy Oil Waterflooding: Simulation and Interpretation Using Pore-Scale Modelling

**DOI:** 10.1371/journal.pone.0169727

**Published:** 2017-01-25

**Authors:** Mohamed Regaieg, Steven Robert McDougall, Igor Bondino, Gerald Hamon

**Affiliations:** 1 Institute of Petroleum Engineering, Heriot Watt University, Edinburgh, United Kingdom; 2 Geoscience Research Centre, Total E&P, Aberdeen, United Kingdom; 3 Total E&P, Pau, France; University of Glasgow, UNITED KINGDOM

## Abstract

Although thermal methods have been popular and successfully applied in heavy oil recovery, they are often found to be uneconomic or impractical. Therefore, alternative production protocols are being actively pursued and interesting options include water injection and polymer flooding. Indeed, such techniques have been successfully tested in recent laboratory investigations, where X-ray scans performed on homogeneous rock slabs during water flooding experiments have shown evidence of an interesting new phenomenon–post-breakthrough, highly dendritic water fingers have been observed to thicken and coalesce, forming braided water channels that improve sweep efficiency. However, these experimental studies involve displacement mechanisms that are still poorly understood, and so the optimization of this process for eventual field application is still somewhat problematic. Ideally, a combination of two-phase flow experiments and simulations should be put in place to help understand this process more fully. To this end, a fully dynamic network model is described and used to investigate finger thickening during water flooding of extra-heavy oils. The displacement physics has been implemented at the pore scale and this is followed by a successful benchmarking exercise of the numerical simulations against the groundbreaking micromodel experiments reported by Lenormand and co-workers in the 1980s. A range of slab-scale simulations has also been carried out and compared with the corresponding experimental observations. We show that the model is able to replicate finger architectures similar to those observed in the experiments and go on to reproduce and interpret, for the first time to our knowledge, finger thickening following water breakthrough. We note that this phenomenon has been observed here in homogeneous (i.e. un-fractured) media: the presence of fractures could be expected to exacerbate such fingering still further. Finally, we examine the impact of several system parameters, including core length, wettability and injection rate, on the extent and efficiency of the finger swelling phenomenon.

## Introduction

Many heavy and extra heavy oil reservoirs contain oil that has limited mobility under reservoir conditions and only a small fraction of the oil-in-place can be recovered using the internal energy of the reservoir itself (through solution gas drive). Hence, at the end of primary recovery, a very small fraction of the initial oil has been recovered. Furthermore, many of these reservoirs are relatively thin and are often contacted by overlying gas or underlying water, making thermal recovery methods impractical and/or uneconomic.

As a part of the research effort in this area, several researchers have studied water injection into heavy and extra-heavy oil. The recovery of heavy oil by water-flooding involves the displacement of a highly viscous fluid by one of much lower viscosity and this often results in the occurrence of so-called viscous fingering, which can be highly detrimental for oil recovery. The fingering causes early water breakthrough and, as a consequence, poor sweep efficiency. However, some recent laboratory studies have suggested that, under certain circumstances, water-flooding into extra-heavy oil (with viscosity up to 7000 cP) could actually result in higher than expected oil recovery [1–3]. Moreover, X-ray imaging of fluid fronts during these experiments has highlighted a finger swelling mechanism that has not been previously reported and the causes of this behaviour–as well as the physics involved in such displacements–have not been explained to date. In this paper, we present a pore network modelling (PNM) study that attempts to shed some light into this interesting phenomenon.

Whilst we restrict our attention here to homogeneous, un-fractured systems, it should be noted for completeness that several researchers have studied viscous fingering in the context of fractured media. For example, Haugen et al [4] studied the effect of wettability on flow in fractured rocks and used nuclear tracer imaging and magnetic resonance imaging in their study. At strongly water-wet conditions, the fractures had a minor impact on the ultimate recovery but significantly changed the progression of the water front compared to the unfractured case. At less water-wet or oil-wet conditions, capillary imbibition of water from the fracture to the matrix was reduced and fractures had a major impact on the ultimate recovery and water breakthrough time. Arabeloo et al [5] studied viscous fingering in low interfacial tension displacements involving heavy oil and surfactant in heterogeneous systems comprising layered porous media containing fractures. They found that the rate of finger growth was largely independent of the type of heterogeneity and that the level of bypassed oil decreased linearly with increasing dimensionless distance travelled by the front. Zendehboudi et al [6] performed an experimental study of controlled gravity drainage in fractured porous media and studied the sensitivity to several system parameters. They found that the critical production rate and recovery factor could be correlated to the fracture/matrix permeability ratio and Bond number.

Several attempts to model viscous fingering in homogeneous media have been reported previously in the literature and an early example makes use of Linear Stability Analysis (LSA) [7–10]. LSA gave some interesting results and Peters and Flock [10] were able to define an instability number that could be used to predict the stability of the displacement front. They tested their theory by performing several flooding experiments and verified that viscous fingering occurred only when the stability number was exceeded–at lower values, the displacements remained stable. Although LSA is an interesting mathematical approach, Riaz et al [11] have emphasized that it only gives information about the initial stages of the instabilities and cannot be used to study late phases of finger development. To overcome this shortcoming, other techniques, including statistical modelling methods, have been used to predict the behaviour of viscous fingers at later stages. Examples of statistical modelling techniques include Diffusion Limited Aggregation (DLA) [12] and the Dielectric Breakdown Model (DBM) [13]. However, these models are relatively simplistic. For instance, the model of Doorwar and Mohanty [13] has been used to simulate experiments reported in [2] but the flow regime in their model was dependent on the value assigned to a tuning parameter η. They proposed a correlation for determining η as a function of the viscosity ratio that is somewhat simplistic, as viscous instabilities are complex in nature and sensitive not only to viscosity ratio but also to a range of other rock/fluid parameters, such as capillary number, wettability and interfacial tension as shown by various experimental studies [10,14].

Riaz et al [11] have presented an alternative approach and investigated the possibility of reproducing unstable displacement experiments using continuum models. They concluded that, although the onset of instability can be determined with acceptable precision, such models are not suitable for studying fully developed flows.

Computational fluid dynamics (CFD) simulation techniques have also been used to simulate viscous fingering at small scales. Clemens et al [14] developed a CFD Navier-Stokes simulator in this context. First, images of the porous medium were obtained by a Scanning Electron Microscope (SEM), periodic grids were then constructed from the images of a single unit block, and mass conservation and momentum conservation equations were subsequently solved–the phase saturations could be determined throughout. This simulator was used to simulate micromodel experiments and it successfully reproduced viscous fingering during water and polymer floodings. The model predicted severe fingering for water injection into oil and an improved recovery for the polymer flooding case. However, their model could not reproduce the dendritic structures characteristic of viscous fingering.

The Lattice Boltzmann method (LBM) is another popular CFD technique. Unlike Navier-Stokes simulators, the Navier–Stokes equations are not solved directly: instead, the discrete Boltzmann equation is resolved to simulate the flow of a Newtonian fluid with collision rules. Compared to the conventional CFD models, LBM has the advantage of being simple and effective for modelling flow in small-scale complex geometries [15]. Dong et al [16] proposed a LBM taking into account both wettability and gravity and were successful in reproducing flow experiments in Hele Shaw cells. They analysed the effects of capillary number, viscosity ratio, Bond number and wettability upon viscous fingering and showed that, when gravity is not considered and the fluid viscosities are similar, the displacement is not sensitive to the capillary number. In addition, the model predicted that the areal sweep efficiency decreased with an increase in the viscosity ratio. However, this model could not consider viscosity ratios greater than 5. Recently, Huang et al [17] proposed a lattice Boltzmann model that could handle viscosity ratios ranging from 10^−3^ to 10^3^. This model was used to build a phase diagram qualitatively consistent with that published by Lenormand et al [18]. Finally, we note that Liu et al [19, 20, and 21] have developed a Lattice Boltzmann model that was able to simulate the main flow regimes (capillary fingering, viscous fingering and stable displacement). This model was applied to simulate drainage micromodel experiments of liquid CO2 displacement of water. The simulator was first applied to dual permeability systems, where the LCO2 was found to displace water only within the high permeability zone at low rates, whilst LCO2 breakthrough occurred within the low permeability zone at high rates. The model was also applied to simulate micromodel experiments and the numerical results confirmed the experimental finding–namely that the steady state interfacial length exhibits a linear dependence on the saturation of the non-wetting phase for both favourable and unfavourable viscosity ratios. Zhang et al [22] used the same Lattice Boltzman model to simulate two phase flow with differing densities on two digital cores (sandstone and shale). They found that fingering was exaggerated at high density ratios. Although these simulations exhibited high stability and accuracy in reproducing the experimental observations, their approach was limited to very small samples and only a small part of the micromodel experiments could be simulated, as simulating the entire system exceeded their computation capacity. In summary, whilst CFD simulators are able to broadly reproduce viscous instabilities and offer some insight into the mechanisms involved, they are computationally expensive and cannot presently be applied to core scale systems.

Pore network modelling is now a long-standing technique for the study of various pore scale phenomena that cannot be reproduced or explained by conventional continuum-scale numerical simulators (see, for example, [23–25]) for details of methodologies and various applications). In this approach, the porous medium is modelled as a number of interconnected pore elements representing the void space in the rock matrix. Various multiphase flow models can then be developed and applied to this in silico network model and several authors have used unsteady-state pore-scale simulators to model viscous fingering during immiscible displacements.

One of the first studies to describe such a model was presented by Lenormand et al [18], who developed a dynamic drainage pore network model and used it to simulate corresponding micromodel experiments until injected phase breakthrough. They could reproduce capillary fingering, viscous fingering and frontal displacement regimes for different capillary number and mobility ratio combinations and used these results to build a phase diagram demarcating the three main flow regimes. The largest networks that they used in their simulations consisted of 100*100 nodal junctions and they presented a relatively limited set of simulation results.

Aker et al [26] developed an unsteady state drainage pore-scale simulator and also used it to simulate different flow regime patterns. They introduced a new method that allowed the simultaneous flow of two liquids into one pore and demonstrated that viscous fingering could be reproduced. However, the approach was highly CPU intensive and a 60*80 network was the largest lattice that could be considered; in addition, they only presented results before breakthrough of the injected phase.

Singh and Mohanty [27] performed three dimensional simulations using a dynamic drainage network simulator and studied the effect of capillary number and viscosity ratio on residual saturations. They simulated both pre- and post-breakthrough behaviour but none of the saturation maps presented in the study exhibited finger swelling. Whilst their simulator could produce various displacement regimes, including viscous fingering, the 3D saturation patterns presented could not be verified against experiment (primarily due to the fact that the largest system used in their study was a 30*8*8 network).

Ferer et al [28] focussed upon the crossover between capillary fingering and viscous fingering regimes and presented two dimensional drainage simulations. They used their simulator to reproduce micromodel experiments performed using fluids with an unfavourable viscosity ratio equal to 62.5. A change of the flow regime from capillary fingering to viscous fingering was observed and viscous instabilities were reported as the capillary number increased–agreement was reasonable between the experimental and simulation study.

Hammond and Unsal [29] used a pore-scale model to study the effects of surfactant upon the flowing regimes and, whilst their dynamic simulator could reproduce the main flowing regimes, including viscous fingering, it failed to simulate the finger shapes observed in experimental studies–the simulated instabilities were very thin, straight and did not have a branched form. The largest network that they simulated was a 70*70 lattice.

Despite the fact that most of the pore network modelling studies described above were performed in small networks, they demonstrate the potential of such an approach to simulate viscous fingering phenomena and to reproduce viscous instabilities with similar shapes to those exhibited in laboratory studies. However, none of the previous models was sufficiently computationally efficient to simulate systems at the laboratory core scale and very few have presented saturation maps after breakthrough for unfavourable viscosity ratios cases. In this study we present a pore network model capable of simulating viscous instability in slab experiments at the scale of tens of centimetres and go on to use this to examine finger behaviour post-breakthrough. The dynamic drainage model builds substantially upon an earlier approach developed by McDougall and Sorbie [24] in the context of unsteady-state displacements in laminated media. We begin here with a validation of the new model against a range of published experimental micromodel results and then go on to simulate laboratory-scale water-floods in large Bentheimer sandstone slabs (30cm x 30cm). We are able to find very good qualitative agreement with the full range of observed displacement regimes, including finger thickening after breakthrough at highly adverse viscosity ratios. We stress here that no attempt has been made to history-match any of the experimental behaviours–once the model has been anchored using physical rock fabric parameters (PSD, connectivity distribution), the water-floods are simply initiated using the appropriate laboratory fluid data and experimental flow rates. Finally, we use our approach to study the effects of varying core length, rock wettability, and injection rate upon the finger swelling phenomenon.

## 1. Description of the Pore Network Model

Flow in porous media is generally governed by the competition between viscous, capillary, and gravitational forces and, whilst the corresponding pore scale phenomena completely determine the flow regime, they are not explicitly taken into account by classical reservoir simulators. As a consequence, reservoir simulators are unable to reproduce certain flow behaviours observed experimentally, such as viscous instabilities [11]. The motivation for the present work is to overcome such shortcomings by developing an efficient pore scale modelling technique to simulate slab experiments at the appropriate scale.

In this paper, we describe a newly developed drainage pore network model that has been built to simulate the full range of unsteady state drainage processes associated with water-oil displacements in porous media. Furthermore, this model has been optimized to facilitate the simulation of slab experiments at the scale of tens of centimetres. Importantly, the new model includes an iterative approach to enable capillary pressure to be taken into account efficiently when solving for the global pressure field.

Firstly, a digital rock surrogate is generated that statistically honours measured pore-scale data (pore size distribution, pore connectivity, pore lengths, pore volumetrics, inter alia). The porous medium is modelled as a number of interconnected pore elements (“pores”) representing the void space in the rock and we focus on modelling the underlying pore-scale mechanisms and examine their aggregated impact at the scale of tens of centimetres. Details of the rock surrogate used will be described more fully in Section 3.3.

Once the network has been created, all pores are filled with the defending phase (oil) and injection of the invading phase (water) begins. The network boundaries are considered sealed, except for the inflow and outflow edges, and the simulator offers the possibility of performing constant pressure drop and constant injection rate simulations. The first step in simulating the network flow consists of solving the pressure field, which becomes a more involved task when considering capillary pressure terms in the formulation – this difficulty was overcome by using an iterative secant method described later.

Having resolved the global pressure field, the individual pressure gradients acting across the pore elements are used to compute local flow rates and these are subsequently used to determine the filling events that control changes in fluid saturation. Here, the interface between phases is considered sharp and well defined and a multiple pore filling algorithm is used to update the menisci in each pore at the oil-water interface, making possible the simulation of concurrent menisci movements occurring at high rates (an important consideration when modelling viscous instabilities). We note that the incompressible nature of the fluids used in our simulations and the fact that the pressure equations are based on mass conservation, ensure that we inject the appropriate volume of fluid at each time step.

The time-stepping process is repeated until a stopping condition is reached and this condition allows the user to choose the stage at which the simulation should stop–either after attaining a fixed saturation value, reaching breakthrough, performing a fixed number of steps, or injecting a fixed volume of the invading phase. At each time step, a clustering algorithm is used to identify the defending phase pores that are no longer connected to the outlet and are therefore considered trapped (i.e. unable to be displaced).

### 1.1. Nodal Pressure Solution

After building the pore network, fluid flows are calculated via a Poiseuille-type law that requires knowledge of local pressure gradients: consequently nodal pressures need to be computed in order to know how the fluids move over a given time step.

**Fig 1:**
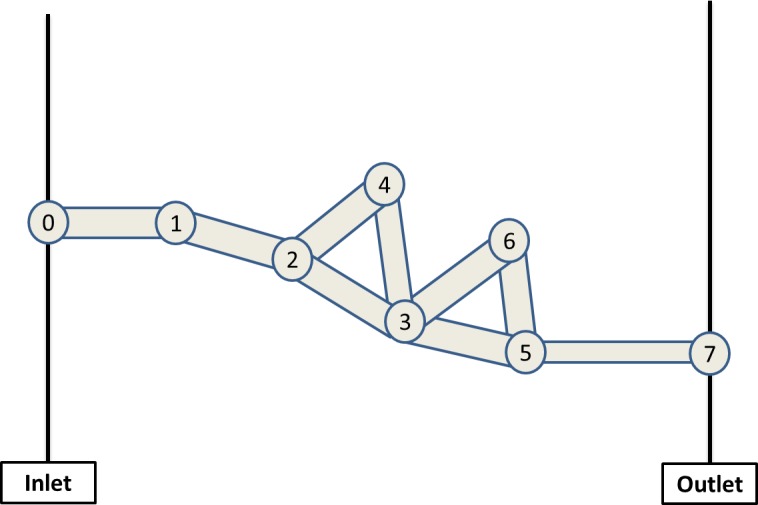
A simple network used to describe the flow of water (white) from inlet to outlet

**Fig 2:**
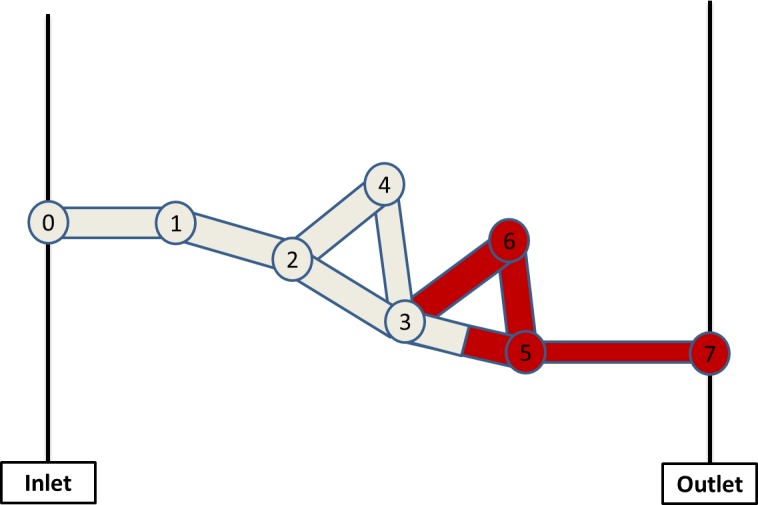
A simple network showing water injection from the left (white), displacing oil (red), which leaves the system via the right-hand pore

In this section, we explain the methodology used to calculate the nodal pressure field within the network and we use the simple networks presented in Fig 1 and Fig 2 to illustrate this. Both examples contain 8 nodes and 9 pores and there is a single inlet node (labelled 0) and a single outlet node (labelled 7).

We will begin with a description of the single-phase flow computations and go on later to extend the approach to two-phase flow. In order to develop a fast and efficient solver, we make the entire formulation non-dimensional and in the following discussion, we use uppercase letters to designate dimensional variables and lower case letters to denote non-dimensional values.

#### a- Single phase flow

Consider first the network shown schematically in Fig 1. Considering mass conservation at a node, we know that (for incompressible flow) flows going in and out are equal, so we can write:

$$\sum_{i,j}Q_{ij}=0$$ (1)

where Q_ij_ is the flow rate from node i to node j.

In single phase flow, the flow rate between two nodes can be computed simply as follows:

$$Q_{ij}=G_{ij}(P_{i}-P_{j})$$ (2)

where P_i_ is the pressure at node i, P_j_ is the pressure at node j and G_ij_ is the conductance of the pore linking nodes i and j, calculated using Poiseuille’s Law for a cylindrical pore element:

$$G_{ij}=\frac{\pi {R_{ij}}^{4}}{8\mu L_{ij}}$$ (3)

where R_ij_ and L_ij_ are respectively the radius and the length of the pore element connecting nodes i and j.

If we consider the pore system in Fig 1 we can write the dimensional form of the mass conservation equation at node 2 as:

$$G_{12}(P_{2}-P_{1})+G_{23}(P_{2}-P_{3})+G_{24}(P_{2}-P_{4})=0$$ (4)

As discussed previously, we wish to work with non-dimensional variables and so we define non-dimensional nodal pressures and non-dimensional conductances as:

$$g_{ij}=\frac{G_{ij}}{G_{s}}$$ (5)

$$p_{i}=\frac{P_{i}}{P_{inlet}}$$ (6)

Here, P_inlet_ is the inlet pressure and G_s_ is a conductance scale defined as:

$$G_{s}=\frac{\pi {L_{s}}^{3}}{8\mu_{s}}$$ (7)

where L_s_ is a characteristic length (taken as the smallest radius in the network) and μ_s_ is the viscosity scale (chosen to be the flowing phases with the largest viscosity).

Dividing Eq (4) by G_s_ and P_inlet_ results in the non-dimensional form of the mass conservation equation:

$$(g_{12}+g_{23}+g_{24})p_{2}-g_{12}p_{1}-g_{23}p_{3}-g_{24}p_{4}=0$$ (8)

Having discussed the mass conservation equation at a typical interior node (node2), we now go on to consider nodes connected to the boundaries: node 1 (connected to the inlet) and node 5 (connected to the outlet). Using the same methodology and considering that the non-dimensional inlet pressure is equal to 1 and the outlet pressure is equal to zero, we can write the following equations:

$$(g_{01}+g_{12})p_{1}-g_{12}p_{2}=g_{01}$$ (9)

$$(g_{57}+g_{56}+g_{35})p_{5}-g_{56}p_{6}-g_{35}p_{3}=0$$ (10)

Notice that the right-hand side of Eq (9) is non-zero whilst the right-hand side of Eqs (8) and (10) are zeros. Now, if we consider the mass conservation equation at every node in the network, solving for the spatial distribution of the pressure field becomes equivalent to solving the linear system:

G*p=q (11)

where G is the coefficient matrix of dimensionless conductances, p is the column vector of dimensionless nodal pressures and q is the column vector of boundary conditions–corresponding to the dimensionless rates at the inlet pores. For the example of Fig 1 the pressure matrices and vectors are the following:

$$G=\left(\begin{array}{cccccc} g_{1} & -g_{12} & 0 & 0 & 0 & 0\\ -g_{12} & g_{2} & -g_{23} & -g_{24} & 0 & 0\\ 0 & -g_{23} & g_{3} & -g_{34} & -g_{35} & -g_{36}\\ 0 & -g_{24} & -g_{34} & g_{4} & 0 & 0\\ 0 & 0 & -g_{35} & 0 & g_{5} & -g_{56}\\ 0 & 0 & -g_{36} & 0 & -g_{56} & g_{6} \end{array}\right),\;p=\left(\begin{array}{c} p_{1}\\ p_{2}\\ p_{3}\\ p_{4}\\ p_{5}\\ p_{6} \end{array}\right)\;and\;q=\left(\begin{array}{c} g_{01}\\ 0\\ 0\\ 0\\ 0\\ 0 \end{array}\right)$$

where we have defined:

$$\left(\begin{array}{c} g_{1}\\ g_{2}\\ g_{3}\\ g_{4}\\ g_{5}\\ g_{6} \end{array}\right)=\left(\begin{array}{c} g_{01}+g_{12}\\ g_{12}+g_{23}+g_{24}\\ g_{23}+g_{34}+g_{35}+g_{36}\\ g_{24}+g_{34}\\ g_{35}+g_{56}+g_{57}\\ g_{56}+g_{36} \end{array}\right)$$

Such a system can be solved using several methods and, in this work, we use Cholesky decomposition to solve the system for 2D networks and a Conjugate Gradient solver coupled with an Incomplete Cholesky Preconditioner to solve the problem for 3D networks.

Whilst the single phase flow model just described can be useful for several applications, such as absolute permeability calculations or tracer injection simulations (dispersion tests), dynamic pore network models are most useful for dealing with multiphase flow problems and these are discussed next.

#### b- Two phase flow

In two phase flow, the flow within a pore situated at the interface separating wetting and non-wetting phases must account for the capillary pressure drop across that interface and so, for a pore element containing both phases, we extend our earlier formulation to:

$$\left\{ \begin{array}{c} Q_{ij}=G_{ij}(P_{i}-P_{j}+P_{c})\;if\;P_{i}-P_{j}>-P_{c}\\ Q_{ij}=0\;if\;P_{i}-P_{j}\leq -P_{c} \end{array}\right.$$ (12)

where the capillary entry pressure is defined using the Young-Laplace equation:

$$P_{c}=\frac{2\sigma cos\theta }{R}$$ (13)

Here σ is the interfacial tension, θ is the contact angle (θ = 180° for a strongly oil wet system) and R is the radius of the pore throat. Notice that (i) the capillary entry pressure is negative for an oil wet system, and (ii) that counter-current flow of oil is not considered.

After writing the non-dimensional mass conservation equation for node 3 (Fig 2) we have:

$$(g_{23}+g_{34}+g_{35}+g_{36})p_{3}-g_{23}p_{2}-g_{34}p_{4}-g_{35}p_{5}-g_{36}p_{6}=C_{s}*g_{35}*\left(-\frac{cos\theta 35}{r35}\right)+C_{s}*g_{36}*\left(-\frac{cos\theta 36}{r36}\right)$$ (14)

where C_s_ is the capillary scale defined as $C_{s}=\frac{2\sigma }{P_{inlet}L_{s}}$,

The right-hand side of Eq (14) now includes a term representing the effect of pore capillary entry pressure, and so the matrix form of the problem now becomes:

$$G*p=q_{b}+C_{s}q_{c}$$ (15)

For the example of Fig 2, the pressure matrices and vectors now become:

$$G=\left(\begin{array}{cccccc} g_{1} & -g_{12} & 0 & 0 & 0 & 0\\ -g_{12} & g_{2} & -g_{23} & -g_{24} & 0 & 0\\ 0 & -g_{23} & g_{3} & -g_{34} & -g_{35} & -g_{36}\\ 0 & -g_{24} & -g_{34} & g_{4} & 0 & 0\\ 0 & 0 & -g_{35} & 0 & g_{5} & -g_{56}\\ 0 & 0 & -g_{36} & 0 & -g_{56} & g_{6} \end{array}\right)\;,\;p=\left(\begin{array}{c} p_{1}\\ p_{2}\\ p_{3}\\ p_{4}\\ p_{5}\\ p_{6} \end{array}\right)\;,\;q_{b}=\left(\begin{array}{c} g_{01}\\ 0\\ 0\\ 0\\ 0\\ 0 \end{array}\right)\;and\;q_{c}=\left(\begin{array}{c} 0\\ 0\\ g_{35}*\left(-\frac{\cos\theta 35}{r35}\right)+g_{36}*\left(-\frac{\cos\theta 36}{r36}\right)\\ 0\\ g_{35}*\left(-\frac{\cos\theta 35}{r35}\right)\\ g_{36}*\left(-\frac{\cos\theta 36}{r36}\right) \end{array}\right)$$

where we have defined:

$$\left(\begin{array}{c} g_{1}\\ g_{2}\\ g_{3}\\ g_{4}\\ g_{5}\\ g_{6} \end{array}\right)=\left(\begin{array}{c} g_{01}+g_{12}\\ g_{12}+g_{23}+g_{24}\\ g_{23}+g_{34}+g_{35}+g_{36}\\ g_{24}+g_{34}\\ g_{35}+g_{56}+g_{57}\\ g_{56}+g_{36} \end{array}\right)$$

q_b_ and q_c_ are respectively the column vector of boundary conditions (corresponding to the non-dimensional rates at the inlet pores), and the capillary pressure column vector (representing the pressure drops across fluid-fluid interfaces).

### 1.2. Coupling injection rate and inlet pressure

Our simulator provides the possibility of performing both constant differential pressure and constant flow rate simulations. Whilst the former are relatively straightforward, the latter adds another level of complexity to the problem, as the dimensional inlet pressure is not known a priori and a scaling is required once a dimensionless pressure field has been calculated. The procedure used to calculate the time-dependent inlet pressure during constant flow rate simulations is described next.

#### a- Single phase flow

Determining the dimensional inlet pressure for single phase flow is straightforward because of the linearity between the inlet pressure and the injection/production rate. Initially, the non-dimensional pressure field is solved (with boundary conditions P_in_ = 1, P_out_ = 0) and the non-dimensional rate at the outlet is computed using:

$$q=\sum_{i\in O}\sum_{j\in {NB}_{i}}g_{ij}(p_{j}-p_{i})$$ (16)

where:

- O = {outlet pore bodies} and NB_i_ = {body j; j is a neighbour of node i};
- g_ij_: is the non-dimensional conductance of the throat connecting bodies i and j;
- p_i_ and p_j_: are the non-dimensional pressures at nodes i and j.

Now, the dimensional rate at the outlet can be written as:

$$Q=G_{s}P_{inlet}q$$ (17)

where G_s_ is the conductance scale defined earlier as $G_{s}=\frac{\pi {L_{s}}^{3}}{8\mu_{s}}$

For constant rate simulations, the dimensional rate Q = Q_target_ is an input to the model and so the dimensional inlet pressure can be found straightforwardly from $P_{inlet}=\frac{Q}{G_{s}q}$.

#### b- Two phase flow

In 2-phase flow, the inlet pressure corresponding to the target rate is once again not known in advance. Furthermore, the relationship between the inlet pressure and the rate at the outlet is no longer linear when capillary pressure is taken into account (as the value of the inlet pressure actually affects the distribution of pores that are available for displacement).

In order to address this issue, an iterative Secant Method is used to find P_inlet_ satisfying Q(P_inlet_) = Q_target_

At each iteration, the following steps are performed:

First, the inlet pressure at the kth+1 iteration is calculated from:

$$P_{inlet\;k+1}=P_{inlet\;k}-(P_{inlet\;k}-P_{inlet\;k-1})\frac{\left(Q(P_{inlet\;k})-Q_{target}\right)}{\left(Q(P_{inlet\;k})-Q(P_{inlet\;k-1})\right)}$$ (18)

Using this inlet value, the pressure field is then computed, the corresponding rate is calculated, closed pores are identified and their conductances are set to zero. The pressure is then re-solved under the new conditions. If newly closed/opened pores are identified, they are closed/opened and the pressure field is calculated once again–the procedure is repeated until all closed/open pores are identified and the rate at the outlet is consistent with the target injection rate.

This method can be made faster by carefully choosing P_inlet k_ and P_inlet k−1_ in the first iteration. One of the starting points is the inlet pressure found in the previous step, which is generally close to the current solution. The second point is found by calculating a fast approximate solution via a modified version of the secant method described previously. Only pores satisfying |P_c_| > P_inlet_ are closed and such pores can be determined before solving the pressure field. As a consequence, we can rapidly calculate an initial pressure solution that represents a good second starting point for the secant method described above. If the starting points for the algorithm are chosen appropriately, the inlet pressure corresponding to the target injection rate, as well as the pressure solution and the consistent distribution of closed/open pores, is generally found after only one or two iterations.

### 1.3. Multiple pore filling algorithm

Once the nodal pressures have been computed, we go on to fill the pores and update the menisci using a multiple pore filling algorithm. First, we loop through the pore elements located at the interface between oil and water and, for every element; a net driving pressure ΔP_net_ is calculated:

$${\Delta P}_{net}=P_{i}-P_{j}+P_{c}$$ (19)

If the net driving pressure is positive, water will be able to invade that pore and the time required to completely invade it is computed as:

$$t=\frac{V_{pore}(1-S_{w})}{{\Delta P}_{net}G_{ij}}$$ (20)

where S_w_ is the water saturation in that pore and V_pore_ is the total volume of the pore.

**Fig 3:**
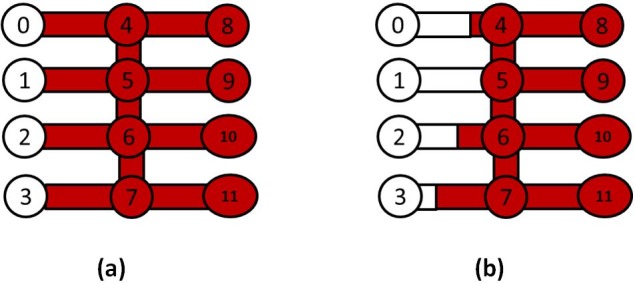
Schematic illustrating the multiple filling algorithm in a case where water (white) invades oil filled pores. (a) represents the pores occupancy before the invasions and (b) shows the fluids distributions after the invasions. Note how the Pore element 1 is completely filled and all the other menisci advance according to Eq (21).

The minimum filling time t_min_ across the entire network of pores is found and the pore characterised by the shortest filling time is completely invaded with water. Menisci are simultaneously updated in all other pores having t > t_min_ (Fig 3) and the water saturation is updated in each pore element and the new water saturation is computed from:

$$S_{w\;new}=S_{w\;old}+\frac{t_{min}{*\Delta P}_{net}*G_{ij}}{V_{pore}}$$ (21)

## 2. Validation of the Model Against Experimental Data

As a first test of the dynamic drainage model, a network was constructed to represent the micromodel experiments described in the ground-breaking paper of Lenormand et al [18].

**Fig 4:**
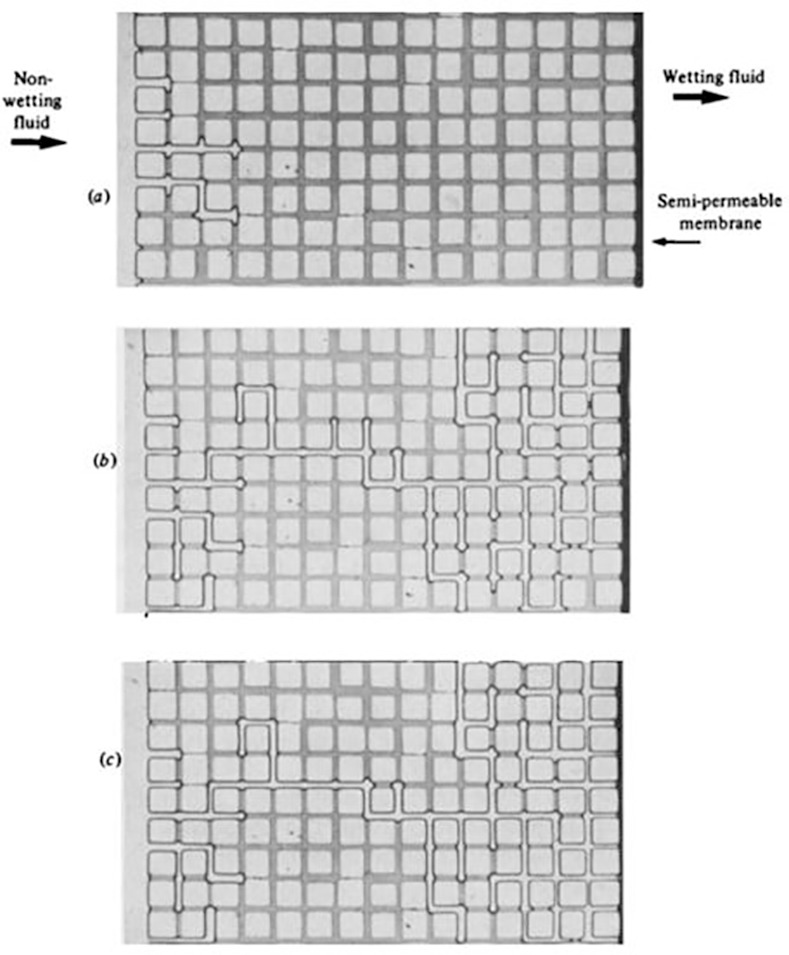
An example of the networks used by Lenormand et al [18]. The non-wetting fluid is injected at the left of the picture [30]. Fig 4. is a reproduction of Fig 7 in Lenormand et al [30].

Lenormand et al [18] fabricated transparent micromodels by means of a moulding technique using transparent polyester resin and a photographically etched mould. Etched ducts with rectangular cross-section were situated inside the resin and this constituted the 2D analogue of the pore space (Fig 4). The micromodels (15cm*13.5 cm) comprised regular etched networks with a coordination number equal to 4 and the bonds had a uniform pore size distribution with radii varying between 115μm and 345μm. A variety of fluid combinations and injection rates were used in order to investigate a number of emerging flowing regimes.

In this part of the paper, we design a network model that mimics the experimental set-up described in the Lenormand paper [18] – the primary aim here is to check if the developed simulator is able to reproduce the same flow regimes as those observed in the laboratory.

As seen in Fig 4 the micromodel topology is very close to that of a bond-only network – there are no large pore bodies surrounded by narrow pore throats–and so our pore element model seems appropriate. The pore radii were assigned stochastically from the reported experimental pore size distributions and the experimental fluid properties and injection rates were used as inputs for the simulations. The shape of the pore elements should not affect the flow regimes in these simulations therefore we use cylindrical pore elements for simplicity. We stress here that there was no attempt to history match the experimental observations–the inputs to the model were simply those reported in the published paper

### 2.1. Simulation of experiments with favourable viscosity ratio

**Fig 5:**
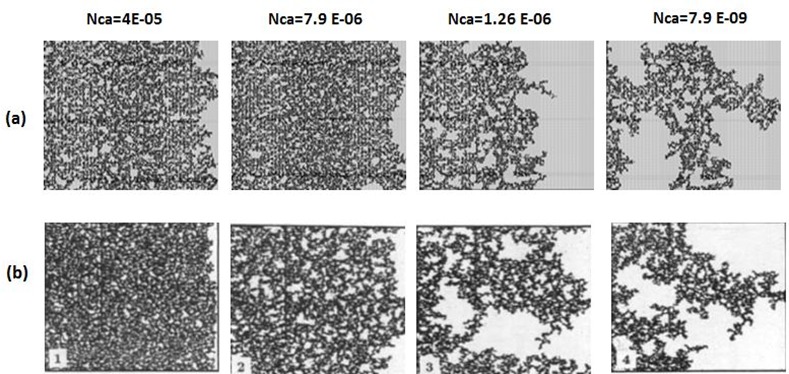
Comparison between (a) simulations and (b) experiments of Mercury (Black) displacing air (white) (viscosity ratio M = 0.0111). N_Ca_ is the Capillary Number and measures the ratio of viscous to capillary forces (see Eq (22)). Fig 5 is composed of images from Fig 10c in Lenormand et al [18]. (Cambridge University Press, reproduced with permission).

**Fig 6:**
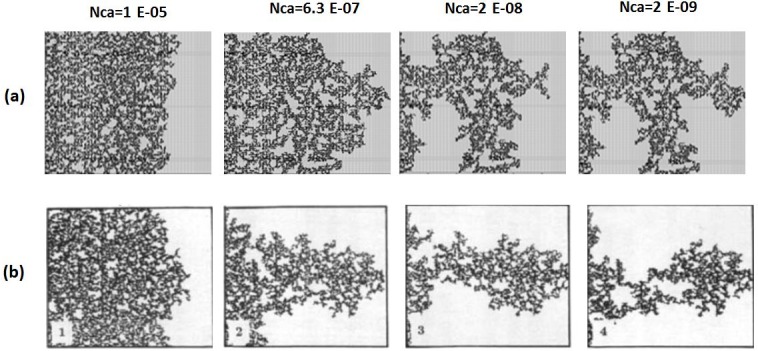
Comparison between (a) simulations and (b) experiments of Mercury (black) displacing hexane (white) (viscosity ratio M = 0.2). N_Ca_ is the Capillary Number (see Eq (22)). Fig 6 is composed of images from Fig 10B in Lenormad et al [18] (Cambridge University Press, reproduced with permission).

**Fig 7:**
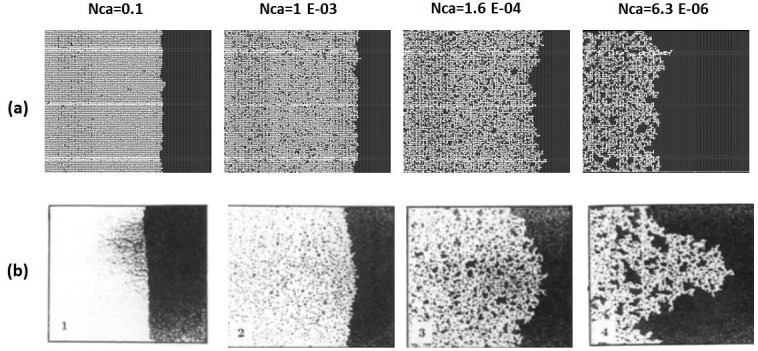
Comparison between (a) simulations and (b) experiments of Glucose solution (white) displacing oil (black) (viscosity ratio M = 0.0098). N_Ca_ is the Capillary Number (see Eq (22)). Fig 7 is composed of images from Fig10d in Lenormand et al [18] (Cambridge University Press, reproduced with permission).

**Fig 8:**
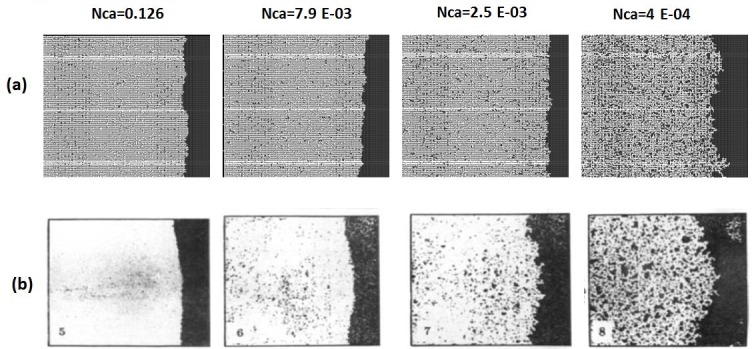
Comparison between (a) simulations and (b) experiments of Glucose solution (white) displacing oil (black) (viscosity ratio M = 0.001). NCa is the Capillary Number (see Eq (22)). Fig 8 is composed of images from Fig 10E in Lenormand et al [18] (Cambridge University Press, reproduced with permission).

Several drainage experiments were performed by Lenormand et al [18] using a favourable viscosity ratio and we now compare qualitatively the saturation maps at breakthrough in both experiments and simulations (Figs 5–8).

At low rates, an invasion percolation like pattern is obtained and this displacement is controlled by the capillary entry pressures of the pores at the interface between the invading and defending fluids – the largest pores having the lowest capillary entry pressure being invaded first. This regime is characterised by invasion events occurring in all directions (even towards the inlet in some cases) and this leads to the emergence of non-wetting phase (water) loops that can trap large quantities of wetting fluid (oil). This behaviour was reproduced by the simulator as shown in Figs 5 and 6. These figures also show that an increase in rate reduces the frequency of loop formation, resulting in less trapped fluid and better sweep efficiency. At high rates, the displacements become stable – the front between the fluids becomes flat and very small volumes of the defending fluid are trapped. It should be noticed that for all the simulations with a favourable viscosity ratio, very good qualitative agreement was found and all the flowing regimes were reproduced by the simulator (Figs 5, 6, 7 and 8). However, we should bear in mind that the network is only statistically equivalent to the experimental micromodel and we do not know the exact radius or spatial location of each physical pore. As a consequence, we should not expect to match the exact displacement paths in the simulations (only the regime).

### 2.2. Simulation of experiments with adverse viscosity ratio

**Fig 9:**
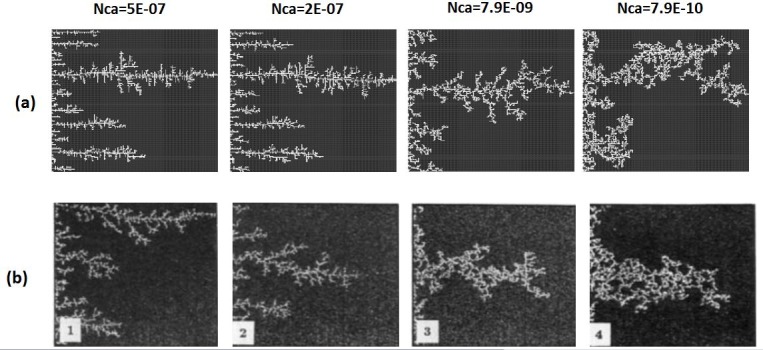
Comparison between (a) simulations and (b) experiments of Air (white) displacing heavy oil (viscosity ratio M = 55555). N_Ca_ is the Capillary Number (see Eq (22)). Fig 9 is composed of images from Fig 10A in Lenormand et al [18] (Cambridge University Press, reproduced with permission).

**Fig 10:**
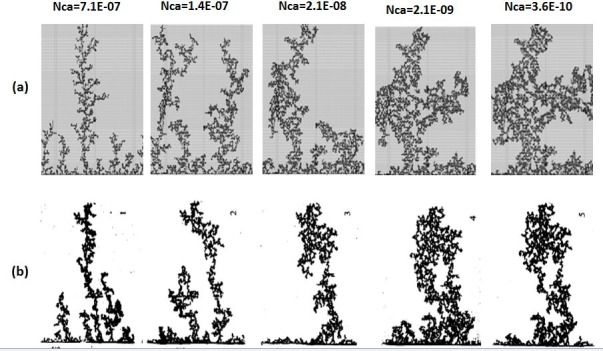
Comparison between (a) simulations and (b) experiments of Mercury (black) displacing oil (white) (viscosity ratio M = 645). N_Ca_ is the Capillary Number (see Eq (22)) Fig 10 is composed of images from Fig 11 in Lenormand et al [18] (Cambridge University Press, reproduced with permission).

**Fig 11:**
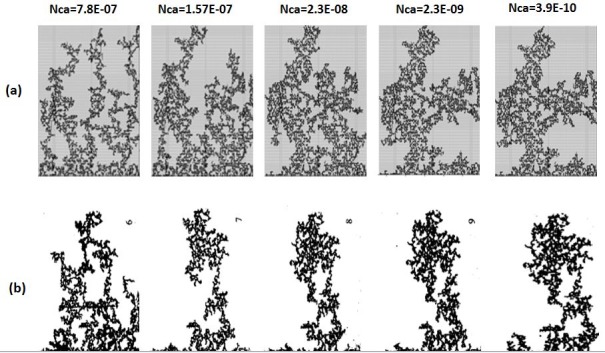
Comparison between (a) simulations and (b) experiments of Mercury (black) displacing oil (white) (viscosity ratio M = 64.5). N_Ca_ is the Capillary Number (see Eq (22)). Fig 11 is composed of images from Fig 11 in Lenormand et al [18] (Cambridge University Press, reproduced with permission).

**Fig 12:**
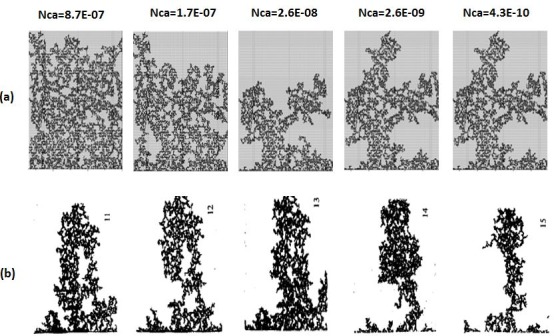
Comparison between (a) simulations and (b) experiments of Mercury (black) displacing oil (white) (viscosity ratio M = 3.6). N_Ca_ is the Capillary Number (see Eq (22)) Fig 12 is composed of images from Fig 11 in Lenormand et al [18] (Cambridge University Press, reproduced with permission).

In the unfavourable viscosity ratio experiments, the flow regimes observed by Lenormand et al [18] at high rates were very different from those observed in the favourable viscosity ratio cases. At low rates, capillary fingering is obtained and an invasion percolation like pattern is observed (with the occurrence of loops trapping large volumes (Figs 9, 10, 11 and 12). However, as the injection rate is increased, the fingers become thinner and sharper until a clear viscous fingering regime is reached at high rates (Fig 9). In this regime, elongated tree shaped fingers are formed that grow towards the outlet, leading to very poor sweep efficiency at breakthrough.

**Fig 13:**
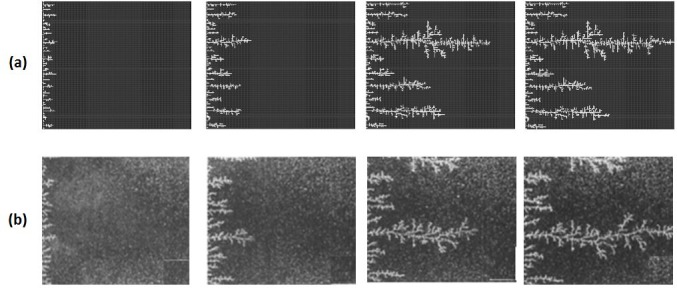
Different stages of displacement of a heavy oil by air after approximately 0.01, 0.03, 0.08 and 0.15 pore volumes throughput: (a) simulation and (b) experiment (viscosity ratio M = 55555). Capillary Number N_Ca_ = 5E-07. Fig 13 is composed of images from Fig 12 in Lenormand et al [18] (Cambridge University Press, reproduced with permission).

Fig 13 shows the different stages of viscous fingering initiation and growth and we notice an excellent qualitative agreement between the simulations and the experiments. In both cases, several fingers were initiated, with some subsequently growing faster and leaving the other instabilities behind. Once again, very good agreement was found with the experiments and all of the observed flow regimes were reproduced over a wide range of viscosity ratios and capillary numbers (Figs 9–12). Note again, however, that we are not expecting to reproduce the fingers at precisely the same locations as observed in the experiments, as we are only reproducing the network stochastically.

In total, our model was tested against 35 micromodel experiments from the literature and excellent agreement has been found in all cases–all of the observed flowing regimes (stable displacement, viscous fingering and capillary fingering) were reproduced and no tuning parameters were required to reproduce the laboratory observations: the parameters reported in the experimental papers were simply used as inputs for the simulation. Although the Lenormand experiments were performed nearly three decades ago, it appears that the only published model that has been able to reproduce the displacement regimes of all the experiments reported in that paper is the one developed for this study [31]. This gives us additional confidence in our in-silico approach and we next go on to apply this framework to the simulation of slab scale experiments of water injection into heavy oil.

## 3. Slab-Scale Study and Finger Thickening

### 3.1. Experimental summary

A number of experiments involving water and polymer injection into extra heavy oil have been reported recently by Skauge anc co-authors using 30cm x 30cm x 2cm slabs of Bentheimer sandstone (details of the experimental procedures adopted can be found in [2]). They used in their experimental study homogenous Bentheimer slabs with a porosity of approximately 24% and permeability of around 2.5 Darcies. The slabs were installed vertically into an X-ray scanner after having been sealed with epoxy resin. The densities of the oil and water were very close to one another and, as a consequence, the displacements were considered to be unaffected by gravitational instabilities.

They installed rail distributors along the inlet and outlet edges of the slabs, with each rail having a central injection/production point and a groove to distribute the fluids across the entire inlet/outlet cross section (images of the apparatus can be found in [2]). Each rock was dried at 80°C; vacuum saturated with 7g/l NaCl brine and then oil was injected. The slab was subsequently aged for 3 weeks at 50°C. Skauge et al [2] have reported two water injection experiments: (i) experiment E7000, using 7000 cP oil with brine injected at the bottom of the slab, and (ii) experiment E2000, where 2000 cP oil was used and injection was performed from the top of the slab. Throughout the following discussion, we will use the term Pore Volume (PV) as a measure of cumulative water injection: one pore volume (1PV) means that the cumulative volume of water injected so far is equal to the total volume of the pore space.

**Fig 14:**
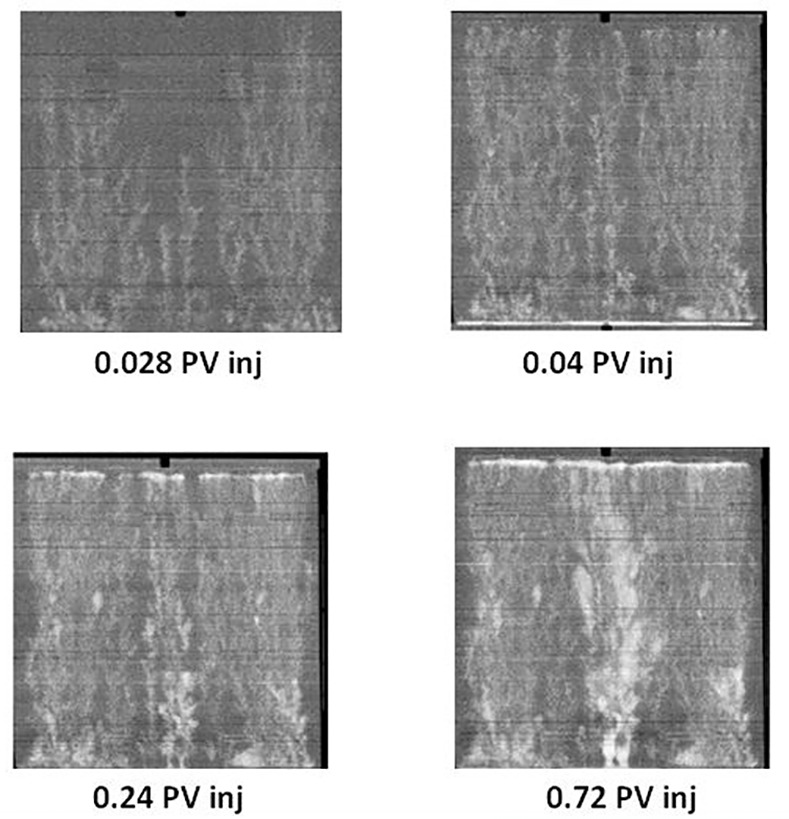
X-Ray images of saturation maps of E7000 experiment for different injected pore volumes [2]. The water (white) is injected from the bottom to displace the oil (black). Note that a pore volume (PV) is a unit used to report the cumulative volume of water injected since the start of a displacement: e.g. 1PV means that the cumulative volume of water injected so far is equal to the total volume of the pore space.

**Fig 15:**
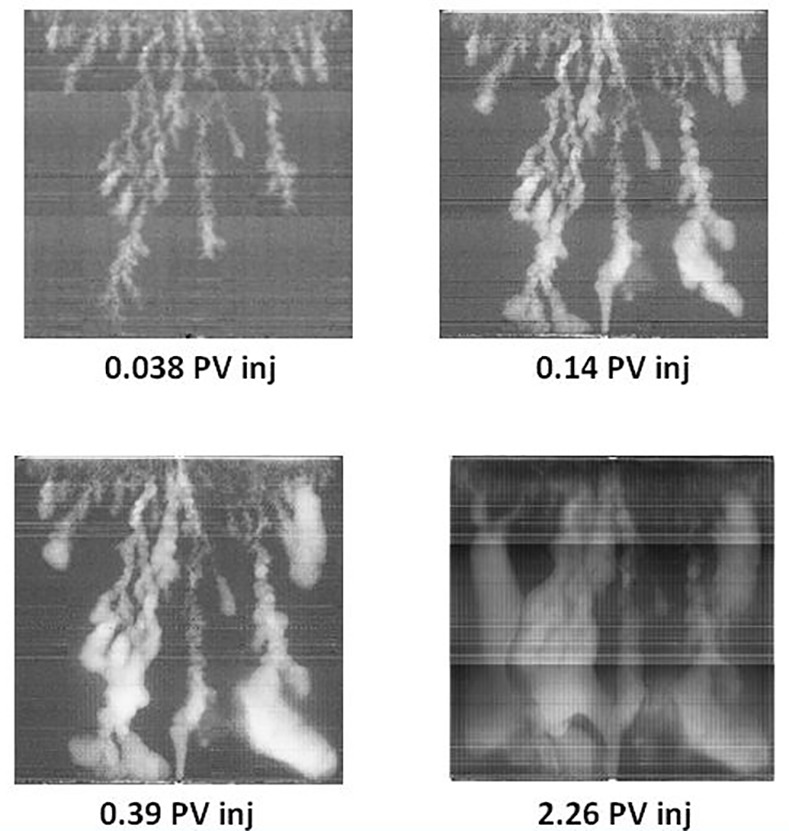
X-Ray images of saturation maps of E2000 experiment for different injected pore volumes [2]. The water (white) is injected from the bottom to displace the oil (black).

They observed the same displacement regime in both experiments during waterflooing (as inferred from X-ray data) and examples of the patterning from both experiments are shown in Fig 14 and Fig 15. Initially, sharp viscous fingers were observed and these grew rapidly towards the outlet edge of the slab. The fingers appeared more dendritic in the E7000 experiment and some instabilities were seen to propagate faster than others, preventing the growth of some of the smaller fingers. Following water breakthrough, fingers throughout the slab appeared to thicken and coalesce, forming broad water channels and leading to an improvement in sweep efficiency. Finger thickening was found to be even more extensive in the E2000 experiment.

**Fig 16:**
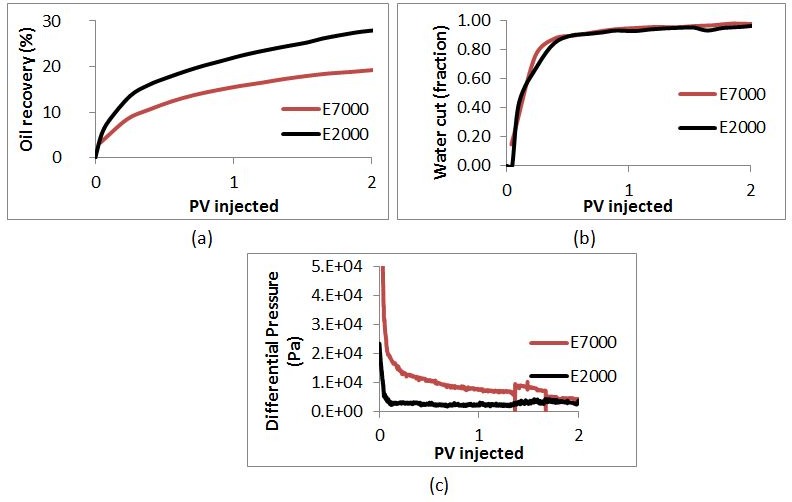
Comparison between (a) Oil Recovery, (b) Water cut and (c) Differential Pressure in E2000 and E7000 experiments as functions of Pore Volumes injected.

Fig 16 shows water-cut data, recovery data, and differential pressure evolution for the E7000 and E2000 experiments [2]. E7000 was characterised by an early water breakthrough after 0.04 PV injected, followed by a rapidly-increasing water-cut that reached 90% after 0.6 PV. After 2.3 PV injected, 20% of the OOIP had been recovered and, at the end of the waterflood (5.1 PV), this had increased to 26.4%–the water cut at this late stage was very high (~99%). Similar early water breakthrough behaviour was observed in the E2000 experiment (0.043 PV injected) but the oil recovery was substantially higher (28.5% of oil had been recovered after 2.3 PV injected versus 20% for E7000).

In order to investigate this intriguing waterflood behaviour, a series of slab scale simulations was performed using rock/fluid data and injection protocols corresponding to those characterising the E7000 and E2000 experiments. We restrict ourselves to large-scale 2D simulations in order to focus on finger morphology and development–consideration of the third dimension is conceptually straightforward but computationally expensive. However, performing 2D simulations requires appropriate scaling of the injection rate and this will now be discussed.

### 3.2. Capillary number in two dimensional simulations

The global capillary number is often used to describe the ratio of viscous to capillary forces acting during two-phase flow in porous media and is generally defined as:

$$Nca=\frac{Q\mu_{displacing}}{\sigma A}$$ (22)

where Q is the injection rate, A is the cross section of the inlet injection face, σ is the interfacial tension and μ_displacing_ is the viscosity of the displacing phase, In 2D simulations, the cross sectional area of the medium (A) is not well defined and so, in order to overcome this shortcoming, the definition in (22) has been adapted to 2D by considering the total flux of invading fluid. By ensuring that the 2D network receives the same flux of fluid as the 3D experiments, their capillary numbers should be equivalent. The equivalent cross section in this case will be computed as A = l_average_ * L_y_ where l_average_ is the average distance between two nodes in the network and L_y_ is the width of the network in the Y direction.

**Fig 17:**
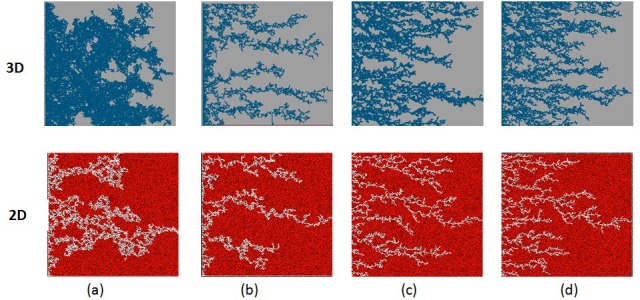
comparison at water breakthrough between 2D and 3D simulations for M = 7000 and matched capillary numbers: (a) Nca = 3.86E-11, (b) Nca = 3.86E-08, (c) Nca = 3.86e-07 and (d) Nca = 1.54E-06.

**Fig 18:**
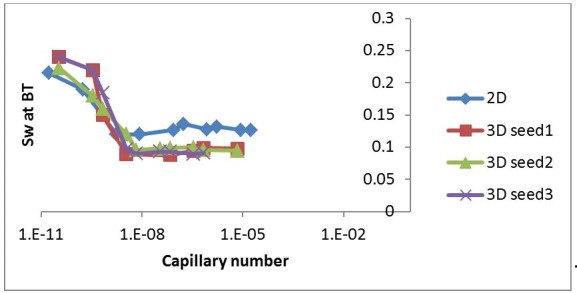
Water saturation at breakthrough versus capillary number. In this figure two sets of simulations are presented: a 2D case (blue) and a 3D case using 3 seeds (red, green and purple). The viscosity ratio was equal to M = 7000 for both sets of simulations.

Fig 17 shows a comparison between 2D and 3D simulations performed using the same viscosity ratio (M = 7000) and a range of injection rates. We see that the same flow regimes were obtained at the same capillary numbers for both 2D and 3D runs. Note that the 3D snapshots are viewed through the top face of the network–i.e. these pictures are actually composites of several 2D layers. Fig 18 shows the corresponding water saturations at breakthrough plotted against capillary number–the water saturation at breakthrough was lower in 3D at high capillary numbers, (corresponding to viscous fingering), which was as expected due to the difference in the dimensionality between the two cases.

### 3.3. Simulation input

The first step in applying our modelling approach to the slab scale experiments consists of building a representative pore network that corresponds to the experimental porous medium. To this end, a network has been extracted from Bentheimer rock images and used to obtain the statistical distributions of the coordination number, pore radii and average distance between two connecting nodes (Table 1).

**Table 1:** Table of parameters used in the simulations.

Contact angle (degrees)	100
porosity	0.24
Average coordination number	3
Minimum coordination number	1
Maximum coordination number	6
Average Radius	15 μm
Rmin	1 μm
Rmax	56 μm
Distribution of Radii	Truncated normal

The final network (1500*1500 nodes) comprises 3,400,000 pore elements. Whilst the simulator is relatively efficient, simulations at this scale still require a long running time, especially if we are interested in post-breakthrough behaviour.

### 3.4. Simulation results

**Fig 19:**
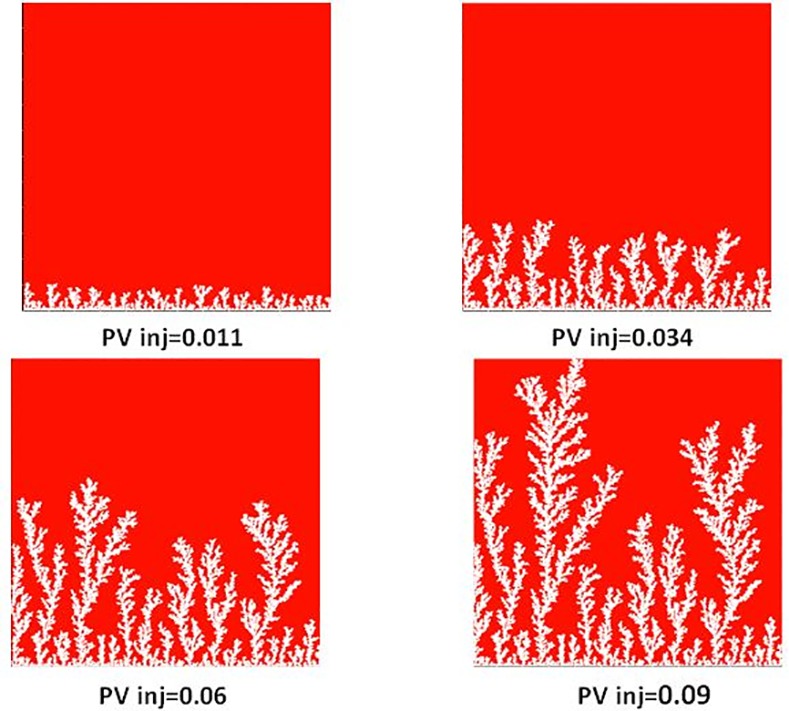
Different stages of viscous fingering initiation and growth in E7000 simulation for different pore volumes injected.

The E7000 and E2000 slab scale simulations performed using our dynamic model exhibit fingering behaviours that are remarkably similar to those observed in the experiments. Initially, several sharp fingers were formed near the inlet of the networks with some subsequently growing faster and inhibiting the development of the shorter upstream instabilities (Fig 19). These fingers had a fern-like structure oriented in the direction of the global pressure gradient.

**Fig 20:**
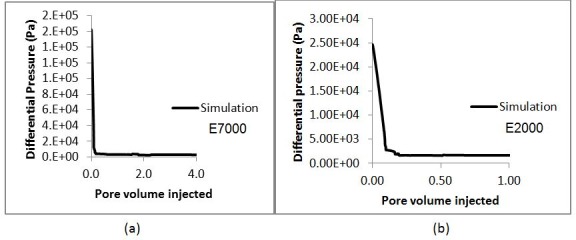
The simulated differential pressure in (a) E7000 and (b) E2000 experiments as functions of Pore Volumes injected.

**Fig 21:**
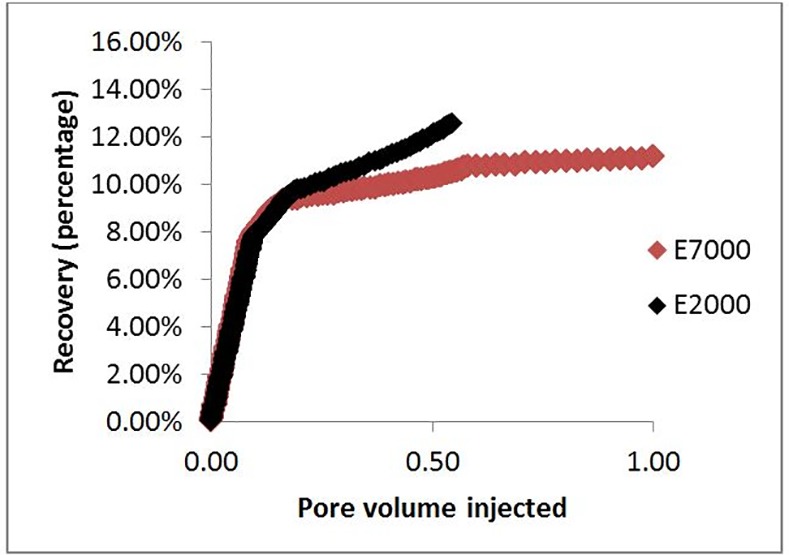
Comparison between the recovery in E7000 and E2000 simulations as functions of Pore Volumes injected.

The simulated differential pressures behaved in a similar way to those observed in the experiments (compare Fig 20 with Fig 16(C)). Initially, the pressure drop was high in both simulations and experiments, then, as the invading fluid advanced, the differential pressure decreased (as expected as a result of the adverse viscosity ratio). We note that the pressure dropped faster in the experiments because of the lower water saturation at breakthrough, which is due to the difference in dimensionality between the experiments and the simulations. After breakthrough, the differential pressures fell further and quickly reached a plateau. Moreover, Fig 21 shows that the recovery was higher in the E2000 simulation, the same behaviour as that observed in the experiments (Fig 16).

**Fig 22:**
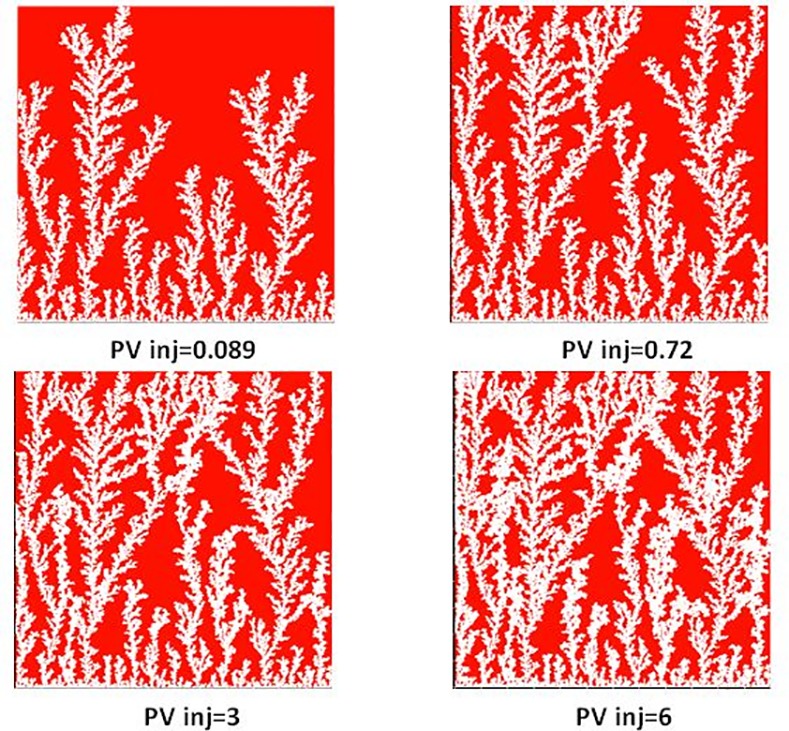
Saturation maps from E7000 simulation for different injected pore volumes. White pores correspond to injected water and red pores contain oil.

**Fig 23:**
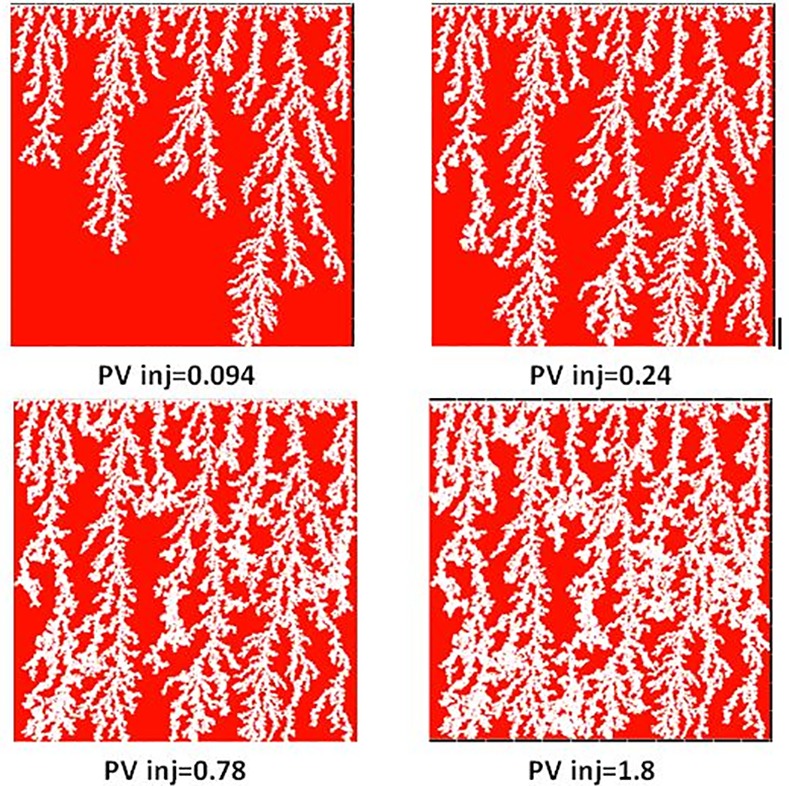
Saturation maps from E2000 simulation for different injected pore volumes. White pores correspond to injected water and red pores contain oil.

**Fig 24:**
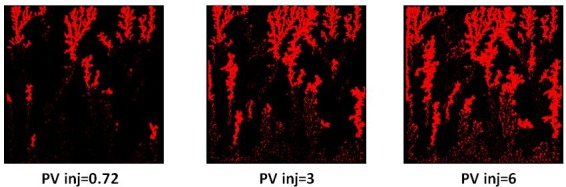
Location of the water invasions that occurred after injected phase breakthrough. These are depicted in red at different stages of the simulation of experiment E7000.

In both E2000 and E7000 cases, sharp viscous fingers were reproduced and, following breakthrough, the simulations exhibited behaviours similar to those observed experimentally (Fig 22 and Fig 23). First, the upstream fingers grew towards the outlet and some degree of finger thickening was observed. This is confirmed by Fig 24 which represents the pore-scale water displacements after breakthrough at different stages of the E7000 simulation. This figure essentially represents the changes between the saturation map at breakthrough and later stages of the displacement. We can clearly see that the new water invasions occurred primarily around the tips of previous instabilities that had not yet reached the outlet. After several fingers reached the outlet, the water started to spread in all directions, resulting in further finger thickening and the emergence of broad water channels as a result of coalescing fingers. Of course, throughout this particular work we must be aware that direct visual comparisons between our 2D simulations and 3D slab experiments can only be qualitative. We also must always bear in mind that X-ray data are essentially 2D composites of 3D scans.

### 3.5. Finger thickening interpretation

**Fig 25:**
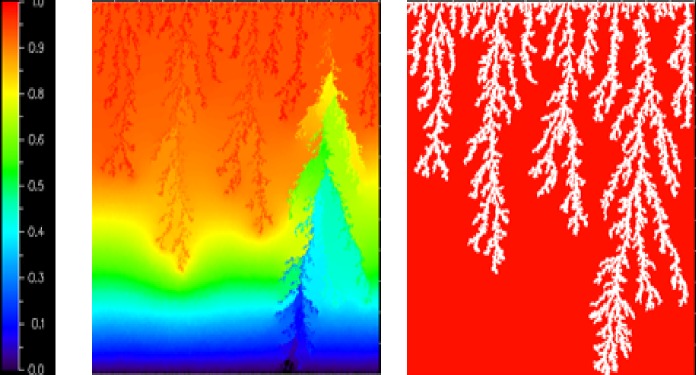
Pressure of the resident fluid in each pore (a) and the saturation path (b) in E2000 simulation at breakthrough of the injected phase. Note that some pores contain water and some pores contain oil–the pressures shown here correspond to the pressure within the resident fluid.

If we examine the evolution of the differential pressure in the E7000 and E2000 simulations (see Fig 20), we notice that it was high at the start of the simulations – hence, viscous pressures were much stronger than capillarity initially, leading to the occurrence of sharp and dendritic fingers. Following water breakthrough, however, the differential pressure dropped across the system resulting in a reduction of the influence of viscous forces and an increase in the importance of capillary forces. Furthermore, when we examine the pressure profile of E2000 more closely at breakthrough (Fig 25), we notice that the highest pressure gradients occurred around the perimeters of fingers situated upstream of the outlet face of the network. This not only accelerated the growth of these instabilities towards the outlet but also caused some degree of finger thickening–a result of the increasing importance of the capillary forces on the displacement. This is confirmed by Fig 24, which represents the area that was swept after breakthrough (the red colour represents the new pores swept post-breakthrough). We can clearly see that the new water invasions occurred primarily around the tips of previous instabilities that had not yet reached the outlet. After several fingers reached the outlet, the inlet pressure dropped further and reached a plateau. At this stage of the waterflood, the viscous pressures that were driving the water towards the outlet became even smaller and so the capillary forces became increasingly influential in determining the subsequent displacement sequence, with pores having a lower capillary resistance more likely to be filled first. Thus, water began to spread in all directions–the fingers thickened further and broad channels emerged as a result of coalescing fingers.

In light of these results, we propose that the finger thickening phenomenon observed experimentally is primarily caused by a drop in differential pressure across the porous slabs through the formation of sample-spanning fingers at breakthrough, resulting in the highest local pressure gradients after breakthrough being associated with the tips of upstream fingers that have not yet reached the outlet. This makes the flow less viscous dominated, resulting in an increased impact of capillary forces and the onset of finger swelling.

In order to have a better understanding of the finger swelling phenomenon, we now go on to examine the impact of varying a number of system parameters on finger development and thickening. In order to reduce computational runtimes, we choose to use 5cm x 30cm simulations (corresponding to 250*1500 networks) for these sensitivities.

### 3.6. Impact of system variables on finger development and thickening

#### a- Core length

**Fig 26:**
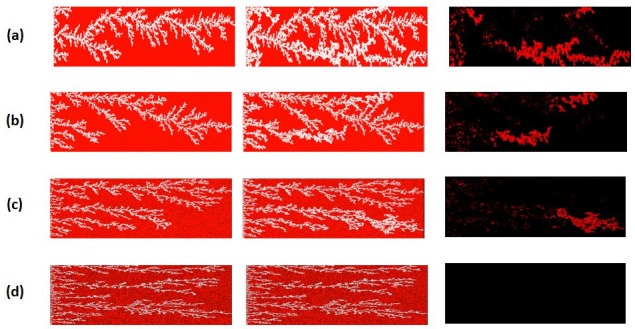
The saturation path at breakthrough (BT) of the injected fluid (left-hand column), the saturation path after 5 Pore Volumes (5PV) of injection (middle column), and the pores displaced between these two times (right-hand column), for different network lengths: 30cm (a), 15 cm (b),7.5 cm (c) 3.25 cm (d). In this set of simulations: Q = 0.005 cc/hr, Nca = 6.9 E-09, theta = 100° and μoil = 7000 cP.

**Fig 27:**
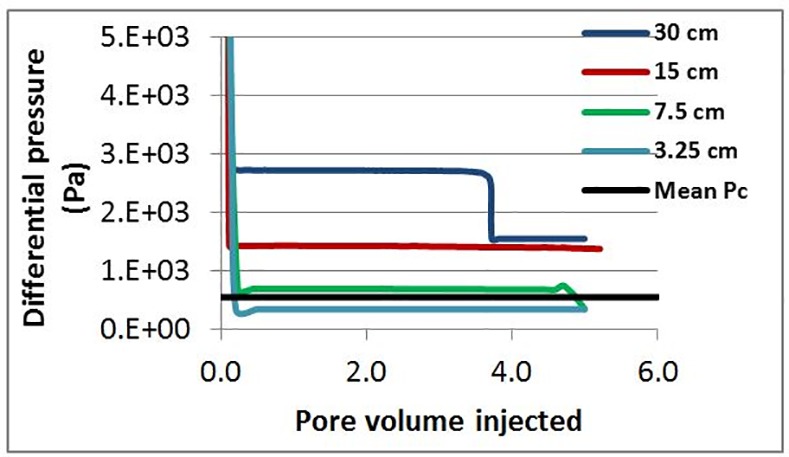
The differential pressure from simulations for different network lengths and mean Pc in the network (calculated using Young-Laplace’s law and the average pore radius in the network). In this set of simulations: Q = 0.005 cc/hr, Nca = 6.9 E-09, theta = 100° and μoil = 7000 cP.

Our first sensitivity focuses on system length and several simulations were performed to investigate the effect of system (core) length on the finger thickening behaviour. The experimental conditions of E7000 were used (using a scaled injection rate of Q = 0.005 cc/hr that matched the experimental flux) and a weakly oil wet condition was assumed throughout (θ = 100°). For network lengths equal to 30 cm and 15 cm, finger thickening was clearly observed (Fig 26) and the plateau differential pressure post water breakthrough remained higher than the mean capillary entry pressure of the underlying network for the duration of the flood (Fig 27) (calculated using the Young-Laplace Eq (13), with the mean pore radius of the network in the denominator).

For a system length equal to 7.5 cm, the pressure drop reached a plateau value very close to the mean capillary entry pressure of the network up until 4.5 PV injection. During this time, the perimeter pores of upstream fingers began to thicken and grow until reaching the outlet. When a second finger reached the outlet, the pressure drop decreased further, falling below the mean capillary entry pressure in the network–shortly after this second pressure reduction, finger swelling ended. An even shorter network (3.25 cm) exhibited contrasting behaviour: in this case, the differential pressure fell below the mean capillary entry pressure in the network as soon as the water reached the outlet and no finger thickening behaviour was observed.

These results suggest that finger thickening occurs when the differential pressure across the system remains higher than the mean capillary entry pressure characterising the pore network and, as a consequence, this suggests that the length of the core plays an important role in the initiation of finger thickening. Moreover, we have demonstrated that short cores may not be able to maintain a differential pressure high enough to cause any finger swelling at all. Such sensitivity to core length could be expected to have major implications for laboratory measurements of displacement efficiency during multiphase flow experiments: an issue that would be of particular concern to the petroleum and hydrology communities

#### b- Wettability

Our previous simulations have been undertaken assuming a weakly oil-wet medium (theta = 100ᴼ), consistent with the experimental conditioning protocol. However, in the absence of quantitative experimental wettability data (i.e. Amott indices or USBM index), it is important to examine the role played by wettability on the extent of finger swelling and oil recovery. To this end, simulations were performed using the experimental conditions of E7000 (Q = 0.005 cc/hr) under two different wettability scenarios: a strongly oil-wet case (θ = 170°) and a weakly oil-wet case (θ = 100°).

**Fig 28:**
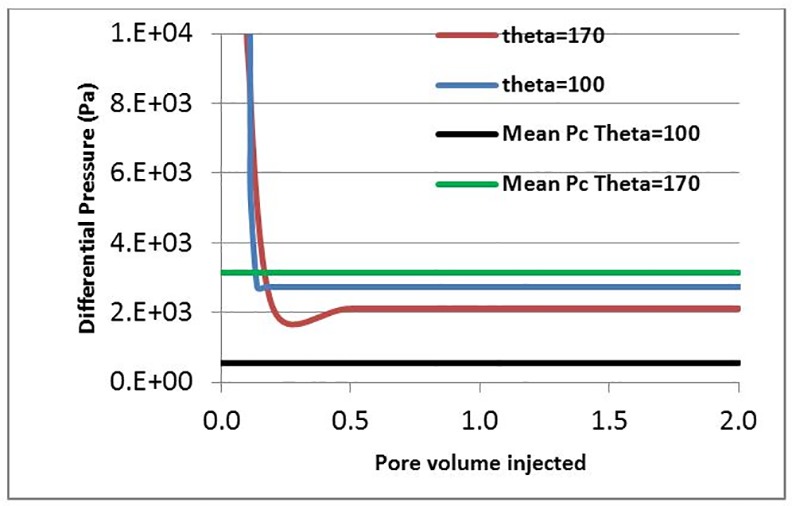
Comparison between the differential pressures in two simulations with different contact angles: θ = 100° (blue) and θ = 170° (red). The mean Pc in the network (calculated using Young-Laplace’s law and the mean pore radius in the network). In this set of simulations: Q = 0.005 cc/hr, Nca = 6.9 E-09 and L = 30 cm.

**Fig 29:**
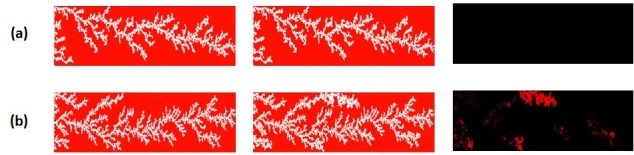
The saturation path at breakthrough (BT), the saturation path after 2PV injection, and the water invasions that occurred between these two times for simulations with different contact angles: θ = 170° (a) and θ = 100° (b). In this set of simulations: Q = 0.005cc/hr, Nca = 6.9 E-09, L = 30 cm and μoil = 7000 cP.

The stabilised value of the pressure drop corresponding to the strongly oil-wet scenario was found to be lower than the mean capillary entry pressure of the network, while for the weakly oil-wet scenario the opposite was the case (Fig 28). Consequently, we found that finger thickening only occurred for the weakly oil-wet scenario (Fig 29)–for the strongly oil-wet case, the saturation path did not change after water breakthrough. This can be explained as follows: a lower contact angle reduces the mean capillary entry pressure of the underlying network and, as a consequence, the differential pressure now lies above this value and so capillary entry criteria can still be exceeded. These results again show that, to have finger thickening, the pressure drop across the system at the plateau stage of the flood should remain higher than the average capillary pressure in the network (varied in this case through the change in contact angle). Again, the implications for experimental studies in this area (particularly core conditioning procedures) are self-evident.

#### c- Injection rate

**Fig 30:**
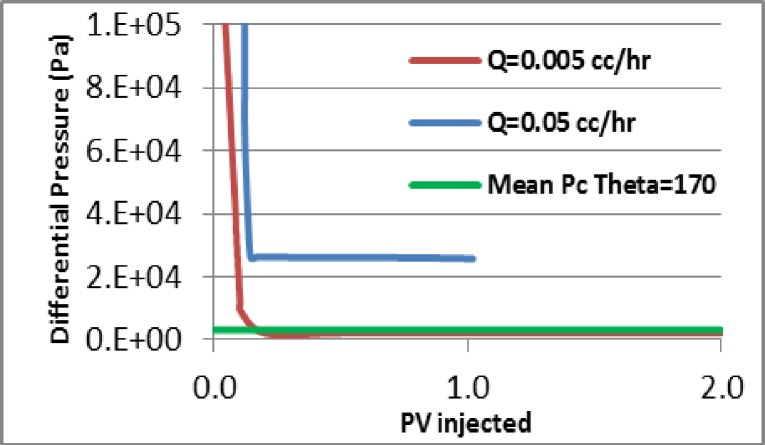
Comparison between the differential pressures in two simulations with different injection rates: Q = 0.005 cc/hr (a) and Q = 0.05 cc/hr. The mean Pc was calculated using the average pore radius in the network. In these simulations θ **= 170° and L = 30 cm.**

**Fig 31:**
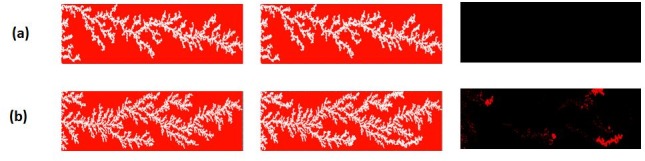
The saturation path at breakthrough (BT), the saturation path after 1PV injection, and the water invasions that occurred between these two times for simulations with different injection rates: Q = 0.005 cc/hr (a) and Q = 0.05 cc/hr. In these simulations θ = 170°, L = 30 cm and μoil = 7000 cP.

As a final investigation, two simulations were performed using the rock/fluid conditions of E7000 at two different injection rates Q = 0.005 cc/hr and Q = 0.05 cc/hr (the former corresponding to the scaled experimental rate). The wettability scenario considered was a strongly oil-wet case (θ = 170°). We found that the simulated pressure drop for the low rate simulation fell below the mean capillary entry pressure in the network (Fig 30), whereas for the high rate it remained higher than the mean capillary entry pressure throughout. Fig 31 shows that the phenomenon of finger thickening only occurred during the high rate simulation, in agreement with our earlier hypothesis.

## Conclusions

Many heavy and extra heavy oil reservoirs contain oil with limited mobility at reservoir conditions and only a small fraction of the oil in place can be recovered using the internal energy of the reservoir (i.e. via primary recovery). Furthermore, many of these reservoirs are relatively thin and are contacted by overlying gas or underlying water, making thermal EOR methods impractical and/or uneconomic. Consequently, water/polymer injection represents interesting alternatives in such situations and understanding the mechanisms involved represents an important step in optimizing recovery.

In this paper, an unsteady state pore-scale drainage model has been developed and applied to a range of experimental studies, from two-phase glass micromodel experiments to water-oil displacements in large-scale sandstone slabs. The model employs an iterative approach to enable capillary pressure to be taken into account when solving for the global pressure field, and an efficient secant method has been developed that allows us to consider time-dependent pressure gradients during fixed injection rate simulations.

The model has been compared against 35 micromodel experiments from the literature and excellent agreement has been found in all cases. All of the observed flowing regimes (stable displacement, viscous fingering and capillary fingering) were reproduced, as well as the transitions between them. Furthermore, no tuning parameters were required to reproduce the laboratory observations: the parameters reported in the experimental papers were simply used as inputs for the simulation

The model has subsequently been used to investigate slab-scale waterfloods of extra heavy oil. These experiments used 30cm x 30cm x 2cm slabs of Bentheimer sandstone (see Skauge et al [2]) and we have been able to construct pore-scale models that honour the experimental pore-scale characteristics and slab dimensions. This surrogate Bentheimer network (1500*1500 nodes) comprised 3,400,000 pores which, to our knowledge, is one of the largest networks that has been used in published dynamic pore network studies.

During the slab-scale waterflooding simulations, several sharp fingers were initially seen to develop near the inlet of the network. Some fingers became increasingly unstable, grew rapidly, and inhibited the development of the shorter instabilities upstream. Whilst fingers were highly dendritic prior to water breakthrough, upstream instabilities were seen to thicken and merge, forming broad water channels after breakthrough and increasing oil recovery. This behaviour was analogous to that observed during the laboratory studies and the finger swelling phenomenon is reproduced here for the first time to our knowledge.

We propose that the finger thickening behaviour is due to a rapid change in the global differential pressure after breakthrough and to the fact that the highest pressure gradients after breakthrough are found primarily around the tips of upstream fingers. We have demonstrated that this phenomenon only occurs when the pressure drop at the plateau stage of the flood remains higher than the mean capillary entry pressure characterising the porous medium.

Finally, it has been shown that system length, system wettability, and injection rate are all important parameters affecting the finger thickening behaviour. Indeed, increasing the length of the core, using a higher injection rate and/or having a more neutral wet condition all increase the potential for finger swelling and improved recovery. These results have significant implications for the interpretation of associated laboratory data that are often used to inform enhanced oil recovery strategies.

## Supporting Information

S1 File Supporting simulation data used in Fig 18, Fig 27, Fig 28 and Fig 30. Dataset A: Represents the simulated water saturation at breakthrough versus capillary number plotted in Fig 18.
Dataset B: Represents the simulated differential pressure for different networks lengths and the mean Pc in the network plotted in Fig 27.
Dataset C: Represents the simulated differential pressure for different contact angles and the mean Pc in the network plotted in Fig 28.
Dataset D: Represents the simulated differential for different injection rates and the mean capillary pressure in the network plotted in Fig 30.
(XLSX)
